# Analysis of Cattle Foot Lesions Recorded at Trimming in the Southwest of England

**DOI:** 10.3390/ani15060829

**Published:** 2025-03-13

**Authors:** Nick Britten, Nicola Blackie, Jon Reader, Richard E. Booth, Sophie Anne Mahendran

**Affiliations:** 1 Royal Veterinary College, Hatfield AL9 7TA, UK; nblackie@rvc.ac.uk (N.B.); rbooth@rvc.ac.uk (R.E.B.); smahendran@rvc.ac.uk (S.A.M.); 2 Synergy Farm Health, Rampisham Down DT2 0HS, UK; jon.reader@synergyfarmhealth.com

**Keywords:** cattle, lameness, welfare

## Abstract

Background: The UK has a high incidence of lameness in cattle, which is costly in terms of economics and welfare. Most causes of bovine lameness originate in the foot but there are several different conditions causing lameness. Quantifying the relative prevalence of different lameness causing lesions allows for the focus of preventative measures and research on the most common conditions. Methods: This study analysed trimming records from 23 professional foot trimmers working in the Southwest of England. A total of 97,944 recorded lesions over a 5-year period were analysed for lesion identity, lesion severity, repeat presentation, foot affected, claw affected and whether they were from dairy or beef cattle. Results: The most frequently recorded lesions were digital dermatitis (32%), white line disease (21%), sole ulcer (19%) and sole haemorrhage (13%). White line disease had the highest percentage of lesions requiring veterinary attention and most frequently re-presented. Most lesions were in hind feet and there was a small but significantly greater number recorded in right feet. Beef cattle had a higher percentage of digital dermatitis and lower percentage of sole ulcer compared with dairy cattle. Conclusions: Digital dermatitis was the most common foot lesion of all cattle types. Most feet with lesions only appeared in the data set once, suggesting broadly that foot trimming was largely effective at resolving new foot lesions. More white line lesions were re-presented, along with having more severe grades compared with other lesions. Therapeutic trimming of chronic lesions appeared to be less successful, with re-presentations, on average, every 93 days, compromising welfare for extended periods, and requiring consideration of different veterinary treatment options.

## Simple Summary

Lameness in cattle predominantly originates in the foot but is caused by several different conditions. By analysing 97,944 lesions recorded by professional foot trimmers examining cattle feet, we found that the most common lesion was digital dermatitis, followed by white line disease, then sole ulcer and sole haemorrhage. Most feet with lesions were hind feet and significantly more right feet were recorded with lesions than left feet. White line disease was the most severe lesion recorded and most often required repeated treatment. The pattern of foot lesions was similar in beef and dairy cattle. We suggest future efforts at preventing lameness focus on digital dermatitis and white line disease as the most common lesions.

## 1. Introduction

Lameness in cattle is a clinical sign caused by a variety of disease processes [1], all inducing pain, hyperalgesia and compromising welfare, but with many of the causes able to be mitigated by timely treatment [2,3]. Foot lesions are the most common cause of lameness in cattle, but the presence of lesions is poorly correlated with lameness [4]. Some foot lesions are always associated with lameness and are termed ‘alarm lesions’ [5]. The identification and treatment of foot lesions, particularly alarm lesions, is crucial to maintaining cow welfare, production and environmental sustainability [1,6,7]. Despite its high importance, the global mean prevalence of lameness in the last 10 years (21.5%) is not appreciably different to the preceding 20 years (24.3%) [8]. Multi-herd studies published between 1993 and 2023 report a mean within-herd prevalence of between 5.4% and 45%, with a range of 0–88% lameness between individual herds [8]. In British dairy cattle a meta-analysis found the pooled prevalence from 1990 to 2019 to be 29.5%, with an incidence of 30.9 cases per 100 cows per year [9]. When estimating the prevalence of different lameness causing lesions, foot trimming records can be a strong source of data despite the bias created through the selection of the animals presented for foot trimming [9]. The estimated incidence rate for the most frequently reported lesions according to meta-analysis was 75.2% for white line disease, 53.2% for sole ulcers and 53.6% for digital dermatitis [9].

The classification of sole lesions differs between authors as sole haemorrhage can be considered a milder clinical sign of corium injury, the same pathological process that results in sole ulcers [10,11,12]. In previous analyses of foot trimming records from the UK and Ireland, sole ulcer and sole haemorrhage (reported either separately or together), have been the most prevalent (33–43% of all lesioned cows) [13,14,15]. When considered separately, sole ulcers are consistently the most prevalent lesion in England and Wales, while in Ireland sole haemorrhage is reported as being five times more prevalent than sole ulcers [13,14]. Whilst the differences between regions of Great Britain appear modest, the differences in reported prevalence between Great Britain and Ireland are large [13,14], which may indicate that farming system is an important factor in the prevalence of different foot lesions, as Irish farms are more typically seasonal pasture-based systems while grazing is declining in Great Britain [16]. Considering other lesions, Murray et al. [10] and Somers and O’Grady [11] both reported white line lesions as the second most prevalent (22–55%), while Reader and Burnell [12] reported that infectious causes (30–35%) were more prevalent than white line lesions (23–26%).

In addition to identifying lesions, foot trimming records allow for an evaluation of the limbs and claws affected. Hind feet are reported as being more frequently affected by UK authors, though the percentage ranges from 82 to 92% [13,14,15]. Likewise, UK authors agree that the lateral claw is most frequently affected in the hind feet and the medial claw in the fore feet [13,14,15]. The foot and claw distributions of lesions are important as they inform foot trimming practices, where the goal is to redistribute pressure away from vulnerable areas of the foot [17]. High lesion prevalence in areas which are deliberately caused to bear more load would suggest the need for adaptations to current foot trimming practices. Most of the published literature regarding lesion prevalence and the claw distribution of lesions focuses on dairy animals [8,14,15,18,19]. Where lesions are reported in beef animals, authors do not agree on whether sole ulcer or white line disease is more prevalent and whether there is a high proportion of infectious cases [20,21,22]. UK foot trimmers report having a low beef caseload and reports are of far fewer animals than dairy-based studies [20,21,22]. UK data directly from foot trimming records are lacking for beef animals and any possible evaluation on beef trims would be a valuable starting point in terms of evidence.

The present study was conducted in the Southwest of England, with the main aim being to provide an updated report of the relative prevalence of foot lesions in both dairy and beef cattle from foot inspection records. Previous studies have analysed each trim as an independent data point [13,18], while the present study utilised analysis using cow-foot identity, allowing for the evaluation of repeated trims of the same animal over a sustained period. It was hypothesised that sole ulcers would be the most prevalent lesion of dairy cattle, white line disease would be the most prevalent lesion of beef cattle, and that there would be a greater proportion of lesions in front feet than hind feet.

## 2. Materials and Methods

Data were collected between March 2018 and December 2023 by 23 professional foot trimmers working for a large veterinary practice in the Southwest of England (Synergy Farm Health, Dorset). All foot timers held a recognised professional qualification and were audited periodically to ensure consistency. The software VetImpress version 3.44.0 (Farmvet systems Ltd., Magherafelt, UK) was used to record lesions observed on feet, with each record containing the date, farm, unique identifying number of the animal, the foot trimmed and lesion(s) identified, along with their location (medial or lateral claw). None of the farms included in this study had a fully systematic approach to presenting cows for foot inspection. Cows identified as lame were generally selected for foot inspection, regardless of production status, while other foot inspections were carried out routinely at predefined points of the production cycle. The size of the farms prevented the implementation of whole-herd trimming at a single visit and most farms had an all-year-round calving pattern so it was not possible to categorise each visit as either routine or to treat lameness. The reason for each cow being presented was not recorded so it was not possible to determine why a cow was inspected from this data set.

A foot that was examined with no lesion identified was considered a ‘foot inspection’. Foot inspections resulting in the treatment of an identified lesion were considered a ‘therapeutic trim’. Lesions were rated as mild, moderate, severe or requiring vet advice (Table 1) based on the opinion of the trimmer at the time of the trim being recorded. This means ratings were estimates of likely outcome and may differ from actual progress.

Data were extracted from the VetImpress software and compiled. Records were analysed at the date and foot level (and so contained both claws). This meant each cow could contribute up to four records per date, and could be re-presented for multiple foot inspections over time. Records with no unique animal identifiable were excluded from analysis. Foot inspection-only records were not analysed further.

Therapeutic trim records underwent descriptive analysis at the foot and claw level for lesion type, re-presentation number, time between re-presentations, and how frequently lesions were consecutively observed on the same foot. The mean number of days between repeated trims of a foot was calculated, with an adjusted figure that excluded trims >124 days apart. A comparison was made between dairy farms and beef farms.

Mixed effects models were constructed to analyse the distribution of lesions between feet and claws using a generalised linear model of the binomial family with a logit link function. To exclude any effect of persistent lesions, only the first presentation of a foot was considered. For the model assessing the distribution of any lesion type between left and right feet, farm, trimmer and lesion(s) recorded were fitted as random factors, with the intercept representing any remaining difference after controlling for these. Lesion identity was fitted as a random effect to allow for generalisation to a population of all possible foot lesions, including those not sampled in this study [23]. Left feet were coded as 0 and right feet as 1. For the models looking at specific lesion distribution between claws, only feet with a single major lesion were considered. Farm, trimmer and limb affected were fitted as random factors and the intercept represented any remaining difference after controlling for these. Medial claws were coded as 0 and lateral claws as 1. Models were constructed for all feet and then for front and hind feet separately as the distribution of lesions by claw has been shown to differ between front and hind feet [13,14,15]. For each specific lesion type, odds ratios for lesion recurrence were calculated to determine the likelihood of a foot re-presenting with the same lesion at the subsequent trim. The population at risk was all foot records of that trim number and the risk factor exposure was the presence of the same lesion detected during the previous trim in the formula (odds in exposed group/odds in non-exposed group) [24]. Analysis was conducted in Microsoft Excel (Microsoft, Redmond, DC, USA) and RStudio (version 2024.12.0.467) using R version 4.4.2 and the lme4 package [25,26,27].

## 3. Results

### 3.1. Data Set

**Figure 1:**
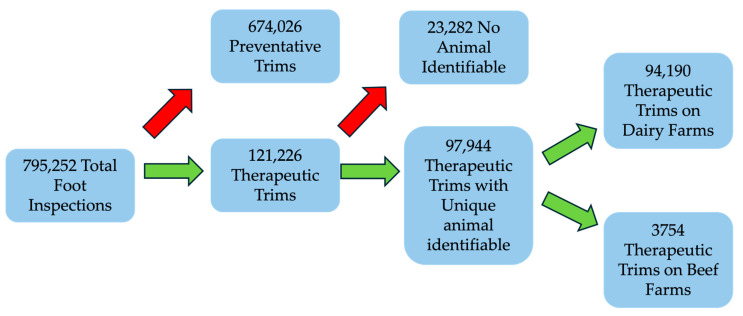
Flow chart of data processing. Red arrows indicate records excluded from analysis. Each record pertains to a single foot inspection event of one foot.

**Figure 2:**
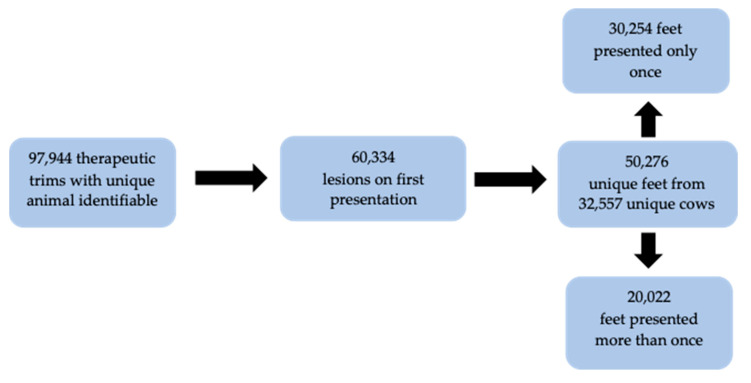
Summary of therapeutic trim records.

A total of 795,252 foot inspection events were recorded. Following exclusions (Figure 1), a total 50,276 feet from 32,557 cows across 346 farms were included (Figure 2). A total of 60,334 lesions were recorded at first presentation. The modal animal presented with a single lesion (83.4%) on a single foot (72.6%). This means that 75.9% (74,299/97,944) of all lesion records were generated by 27.4% (8912/32,577) of cattle. This was predominantly due to multiple feet from the same animal being recorded or the repeated presentation of the same foot. Of the therapeutic trim records, 96% (94,190) were from dairy animals, with the remaining 4% (3754) from beef animals. There were 250 dairy farms and 96 beef farms in the study data set.

### 3.2. Frequency and Percentage of Lesions Recorded at First Presentation

**Figure 3:**
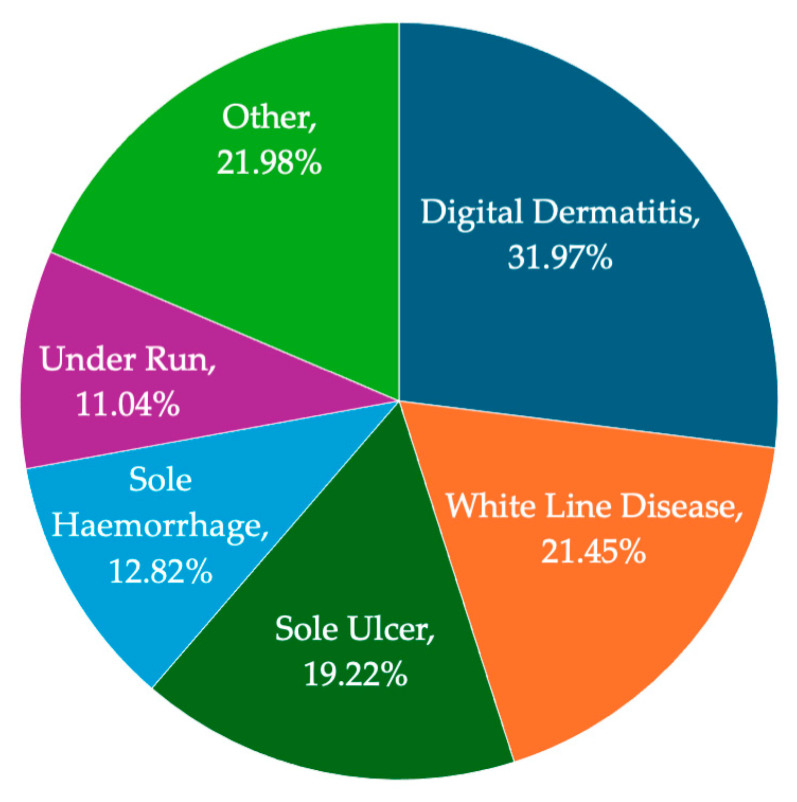
Summary of the percentage of trim records reporting each lesion type. Lesions <10% were amalgamated into ‘other’; details in Appendix A. Note that, as feet could have had more than one recorded lesion, the percentages total more than 100% (*n* = 60,334 lesions).

The percentage of feet with each lesion at first presentation was calculated (Figure 3). The most recorded lesions were digital dermatitis (31.97%), white line disease (21.45%), sole ulcer (19.22%) and sole haemorrhage (12.82%). Digital dermatitis, sole ulcer and white line disease are all ‘alarm’ lesions [5]. Sole haemorrhage is not an alarm lesion but can be considered as part of the same pathology as sole ulcer [10,11]. These four were termed ‘major lesions’ and were the focus of subsequent analyses. A summary of records with all observed lesions can be found in Appendix A. At first presentation, 67.63% (34,002/50,276) of all foot inspection records contained only a single major lesion. The total number of feet with two major lesions at first inspection was 8339 (Table 2). Digital dermatitis and white line disease were the most frequently co-occurring major lesions.

### 3.3. Foot Distribution of All Lesions at First Presentation

A summary of all lesions identified at the first presentation of each foot is given in Table 3. As only the first presentation of a lesion was analysed, any impact of severity leading to a repeated presentation on foot distribution, was eliminated. The percentage of lesions in hind feet (80.8%) was substantially higher than that in the fore feet (19.2%). In hind feet digital dermatitis, sole haemorrhage and sole ulcer were recorded more in the right foot while white line disease was recorded more in the left foot. An analysis of the minor lesions can be found in Appendix A. When controlling for farm, trimmer and recorded lesion, there was a significant (*p* < 0.01) 3% increase in the odds of a foot with a lesion being a right foot (Table 4). Farm, trimmer and lesion identity accounted for very little of the total variance.

### 3.4. Claw Distribution of Major Lesions at First Presentation

Feet with a single major lesion at first presentation (*n* = 34,002) were analysed. Feet with multiple different lesions were excluded as individual lesion location was not recorded. Feet with the same lesion recorded in multiple locations were included. As only the first presentation of a lesion was analysed, any impact of severity leading to repeated presentation on claw distribution was eliminated. Lesions were recorded as being on the medial claw, lateral claw or interdigital area. The areas in which the major lesions were recorded are presented in Table 5. Digital dermatitis would be expected to be recorded in the interdigital area and 94% of records reflected this; however, 5% of records showed digital dermatitis on the medial claw and 8% showed digital dermatitis on the lateral claw. Sole lesions and white line disease should be recorded on a claw, but approximately 2% of the records of sole ulcer, sole bruising and white line disease were in the interdigital area.

Lesion distribution between medial and lateral claws was analysed (Table 6). The interdigital space was not included, as most lesions should not be found there and there is no paired structure to evaluate anatomical symmetry. Digital dermatitis was identified significantly more on the lateral claw than the medial claw across all feet (*p* = 0.03) and in hind feet (*p* = 0.01). Sole haemorrhage was identified significantly more (*p* = 0.02) on the medial claw in the fore feet but on the lateral claw in hind feet (*p* = 0.002). The proportions of sole ulcers and white line disease did not significantly differ between claws in either front or hind feet.

### 3.5. Lesion Severity at First Presentation

The recorded severity of lesions was evaluated (Table 7). Only feet with a single major lesion at first presentation were analysed (*n* = 34,002 records), and each lesion (and therefore foot) received a separate rating of severity. Digital dermatitis had the greatest percentage of lesions evaluated as mild, sole haemorrhage had the greatest percentage of lesions evaluated as moderate and white line disease had the greatest percentage of both lesions evaluated as severe and those requiring vet advice.

### 3.6. Time Between Repeated Trims of the Same Foot

Time between consecutive therapeutic trims of the same foot on the same cow were analysed. Repeated trim frequencies ≥10 presentations were collated into a single category. The number of consecutive trims recorded ranged between 1 and 24 records (Table 8). The mean number of days between each presentation was calculated, along with an adjusted figure which excluded any value >124 days as a ‘new’ lesion, using the ICAR definition [28]. The days between trims appeared consistent around a mean of 155. The adjusted days had a mean of 49.5 and demonstrated stability for the first five presentations followed by a small but consistent reduction in time between trims as the number of re-presentations increased.

### 3.7. Lesion Prevalence and Repeated Therapeutic Trim Number

The percentages of major lesions recorded in feet changed with an increasing number of repeat therapeutic trims (Table 9). Note this analysis did not require the same lesion to be consecutively presented and so includes new cases as well as persistent ones. See Section 3.8 for consecutive presentations of the same lesion on the same foot. Digital dermatitis showed a sharp decline in percentage as trim number increased. Sole haemorrhage increased in percentage from trim one to trim two, then declined with increasing trim number, though it never changed percentage by more than 3%. The percentage of sole ulcers rose to a peak at the third trim then declined with increasing trim number. The percentage of white line disease showed an increasing trend with increasing trim number. The ‘other’ lesions percentage decreased from first to third presentation, before rising with each consecutive trim and becoming the greatest percentage of lesions.

### 3.8. Consecutive Presentation of the Same Lesion

**Figure 4:**
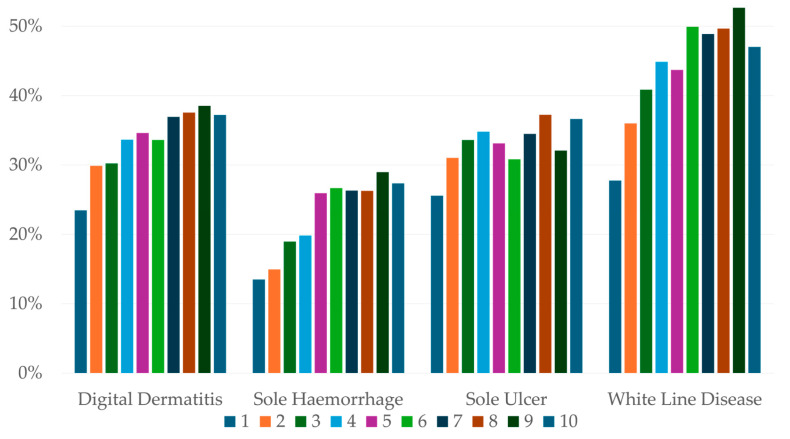
Percentage of lesions re-presented on the same foot by trim number from 50,276 feet from 32,557 cows on 346 farms.

Repeat trim records for the same feet were examined to determine re-presentation rates for the same lesion on the same foot at consecutive therapeutic trims (Figure 4). This eliminates new cases and provides a better estimate of lesion persistence. For the major lesions, around 75% of feet were not re-presented. The percentage of feet with the same lesion at the second trim was low, and is shown in ascending order as follows: sole haemorrhage (13.5%), digital dermatitis (23.5%), sole ulcer (25.6%) and white line disease (27.8%). The percentage of lesions that were re-presented generally increased with trim number for all major lesions.

Odds ratios were calculated for the occurrence of the same lesion on all re-presentations of the same foot at therapeutic trims (see Appendix B for full table). A foot with digital dermatitis had a 55–60% chance of having had the same lesion at the previous trim. Sole ulcer cases had a 48–59% probability of having had a sole ulcer on the previous trim. Sole haemorrhage consistently had the lowest percentage of repeat presentations (28–45%) while white line disease consistently had the greatest percentage of repeat presentations (62–72%).

### 3.9. Differences Between Dairy and Beef Farms

**Figure 5:**
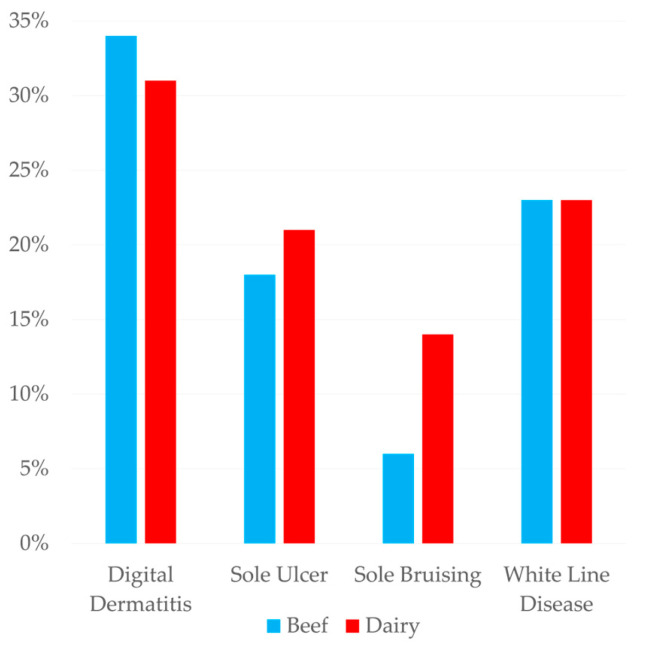
Percentages of feet with major lesions (50,276) on 96 beef and 250 dairy farms.

**Figure 6:**
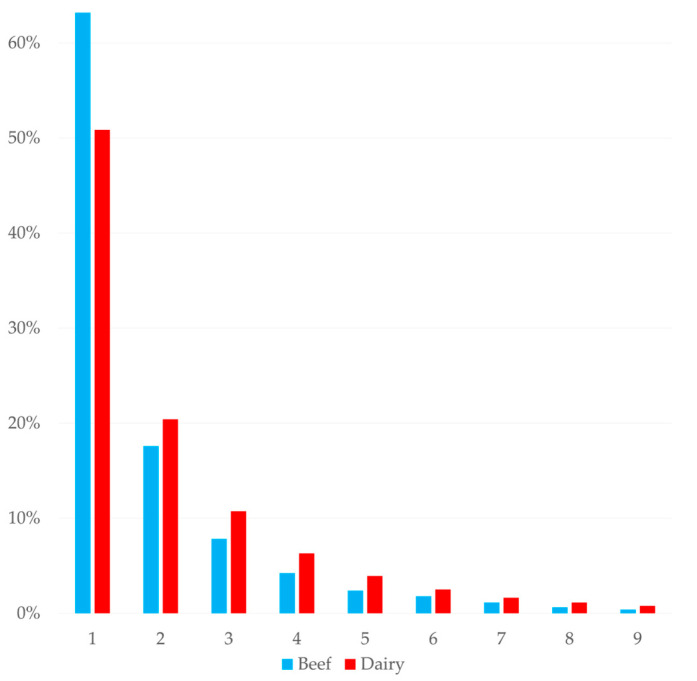
Percentage of each trim number of all trim records (97,944) on 150 dairy and 96 beef farms.

Beef farms (*n* = 96) provided 3754 foot records and dairy farms (*n* = 250) provided 94,190. Comparisons between the two are shown as percentages of the total for each farm type. The percentage of each foot trimmed was very similar between dairy and beef animals (Table 10), though the percentages of major lesions did vary between the two (Figure 5). Digital dermatitis made up a higher percentage of lesions in beef cattle (34%) compared with dairy cattle (31%). In dairy cattle, sole ulcers (21%) and sole haemorrhage (14%) made up a greater percentage of lesions than beef cattle (18% and 6%, respectively). The percentage of white line disease recorded in both systems was 23%. The mean number of times a foot was seen in a dairy animal was 2.3 compared with 1.9 in beef and the distribution of trims per foot was more skewed toward lower numbers in beef (Figure 6).

## 4. Discussion

The purpose of this study was to assess the relative prevalence of lesions recorded at foot inspections of cattle in the UK, the distribution of lesions between feet and claws, the change in lesions with repeated inspection and the differences in lesion patterns between beef and dairy cattle. Whilst lameness was not recorded in this study, the presence of foot lesions would be expected to correlate closely with lameness, particularly ‘alarm’ lesions [5]. Assuming that an inspection without lesions denoted a non-lame animal and a therapeutic trim a lame animal, the estimated lameness prevalence would be 15.24%, lower than previous UK estimates but within the reported range [8]. This is likely an underestimate of the true lameness prevalence as not all lame cows are treated by the foot trimmer—cases treated by farm staff or the veterinarian, those resolved without treatment or those culled would not have appeared here—though not all cows on the farms in this study were presented for foot inspection. The detection of lameness is poor, with veterinarians reported as having an 18% sensitivity in detecting foot lesions with locomotion scoring [4] and farmers only being aware of around 1/3 of those [29].

Despite the time gap, the relative prevalence of foot lesions in this study was very similar to that reported by Murray et al. [10] in Great Britain in 1996. In the present study, the white line disease (WLD) prevalence was within 1% of that shown in the work of Murray et al. [10], but there was a 4.8% increase in sole haemorrhage (SH), a 3.8% decrease in sole ulcer (SU) and a 24% increase in digital dermatitis (DD) [13]. This similarity in lesion prevalence over time may just reflect the lack of change in overall lameness prevalence [8], but is disappointing given the known negative impact that lameness has on both economics and welfare. The present study’s slight increase in SH and slight reduction in SU does suggest that the increasing use of preventative foot trimming may be reducing the progression of haemorrhage to ulceration [11], but more research in this specific area is required.

The increase in DD may be linked to a general move in UK dairy systems away from grazing to increased housing [16]. Housing can increase environmental infection pressures, which may explain why DD appears to have become so much more prevalent. A Swiss study also found DD to be the most prevalent lesion at the cow level, with lower prevalences in more extensive systems [19]. At the herd level, effective foot bathing is key to control, with blitz therapy a possibility in some instances [30,31]. Although individual animal cure is difficult to achieve, treatment protocols using antibiotic and non-antibiotic products alongside bandages have reported success [32,33,34]. Given the relative ease of instigating routine foot bathing in dairy herds (compared with individual animal treatments), this offers the industry an opportunity to prevent the leading cause of lameness in UK cattle with relative simplicity.

To the authors’ knowledge, the present study is the first to report on repeated presentations of the same feet over time, with previous authors having used cross-sectional study designs and shorter study periods [14,35,36] or not analysing repeat presentations [13,15,18]. Most feet with major lesions only appeared in the data set once (75%, Section 3.8). Whilst individual cow treatment records were not assessed, nor the level of culling, these study findings might suggest that foot trimming was largely effective at resolving new foot lesions (i.e., at first presentation). This is a similar finding to that suggesting that 78% of lame cows were sound two weeks after treatment and were significantly more likely to stay sound than untreated animals [37]. WLD had the highest rate of feet that re-presented with the same lesion (27.8%, Section 3.8), but this study shows that 72% of white line lesions were not re-presented for a second trim, which likely indicates that they were resolved. Whilst the data set did include routine foot inspections as well at therapeutic ones, the lack of the systematic presentation of cows for inspection made it impossible to determine for certain why a foot was not recorded again.

SH lesions were the least likely to be re-presented with the same lesion, although this may be due to non-resolving SH progressing to SU as well as treatment resolving the first observed lesion [11]. The percentage of DD steadily decreased with successive re-presentations, while the percentage of WLD steadily increased. This appears to align with the ratings of severity, in which DD was shown to have the greatest percentage of mild lesions, while white line disease was rated most severely. It is also known that claw horn lesions cause damage to internal foot structures, predisposing animals to similar lesions in the future [38,39,40]. Over an approximate five-year period, the mean number of days between consecutive trims of the same foot was 155 days (Section 3.6). This suggests many cows presented for lameness at least twice per year. Farm management decisions in scheduling trimming impacted the time between trims in some cases, but the overwhelming majority of farms included in this data set had a monthly trimming frequency or trimmings were more frequent. Trim intervals greater than 30 days suggest that trimming visit frequency was not the limiting factor. It was previously calculated that most lesions do not predispose to further lameness events after 16 weeks (112 days) [41]. By eliminating records more than 16 weeks apart, the time between trims had a mean of 49.5 days (Section 3.6), suggesting the lesions on these repeat trims were related to those seen on the previous trim. This suggests that current lameness treatments are ineffective for more chronic lesions, which must be addressed to ensure cattle welfare is not being compromised for these extended periods of time. There was a trend for shorter presentation intervals as re-presentation number increased. This is likely due to animals with particularly severe lesions being presented more regularly, and may reflect an increased motivation on the part of producers to present animals which were chronically or repeatedly lame for trimming. Although this is vital for the treatment of the individual animal, if trimmers are constantly presented with chronically lame cows, it is likely that the number of preventative trims seen during that visit may be negatively impacted—this may lead to increases in new lameness cases. More research on the most appropriate treatment protocols for chronic lesions are needed, particularly around decision-making regarding performing a more radical trimming of lesions under local anaesthetic or carrying out claw amputations.

Most lesions were found in the hind feet, in agreement with the previous literature [13,15]. The present study found significantly more lesions in the right feet, an observation that was not previously reported. This was after controlling for farm effect and lesion identity. The origin of this distribution is unclear, but better cure rates have been reported in left feet [42], which could lead to more lesions being recorded in the right. The weight distribution of standing cattle has been shown to be balanced between left and right overall, but a consistent difference of around 10% load-bearing in each pair (fore and hind) of limbs was noted, though to which side was not reported [43]. A comparison of weight distribution between left and right during locomotion also shows imperfect symmetry, even in healthy cattle [44]. Whilst, at the individual level, these reported differences are small, it is possible that the cumulative effect of small differences in the weight-bearing of individuals leads to differences in lesion frequency at the population level. Cattle have also been shown to demonstrate consistent lateralised behavioural preferences [45,46,47], including a preference for lying on the left side [48,49], which could cause an asymmetry in pressure and therefore lesion development. Establishing whether this effect is consistent among study populations and, if so, the mechanism behind it may allow for adaptations to cattle housing, handling or footcare practices to mitigate the additional risk.

The biomechanics of normal locomotion in cattle predispose the lateral claw of the hind foot and medial claw of the front foot to sole lesions [11], which was largely reflected in this study. Foot trimming in the UK focuses on relieving force from the lateral claw in the hind feet [17], so this may result in foot trimming preventing SH progressing to SU on the lateral hind claws but not the medial hind claws, as seen in this study. The relative symmetry in claws with WLD is a likely a consequence of the risk factors for the development of this lesion, with ground conditions and poor footing affecting all claws equally. The relative increase in fore feet WLD may be a consequence of the increased bodyweight distribution compared to the hind feet, with scrabbling and pushing leading to more shear forces being exerted through the front feet.

There was evidence of recording errors in the analysis of the location of lesions within each foot. DD could be located on either claw or the interdigital space, but SH, SU and WLD cannot be found in the interdigital space. Despite this, approximately 2% of SH, SU and WLD were recorded as being in the interdigital space (Table 5). As this is consistent across all three lesions, it implies a data entry error rate of 2% generally. Despite being erroneous, these records were included in the analysis as removing them would impact SH, SU and WLD but not DD, and would therefore introduce a systematic bias in favour of DD to the data set. As far as could be ascertained, errors were most likely due to incorrect button presses at input, and so the 2% implied rate would be expected to distribute evenly between lesions and locations and so was preferable to introducing systematic bias.

The percentage of therapeutic trims conducted on beef animals was 3.83% (Table 10), much lower than both the mean and median amount reported in a survey of UK foot trimmers [22]. The percentages of each affected foot in beef cattle were within 1% of the percentages observed in dairy cattle, suggesting that the same mechanisms cause lesions in both systems. Evaluating the features of individual farms was beyond the scope of the present study but this would be a beneficial topic for future work. The lesions recorded in beef cattle were similar in percentage to those in dairy animals, with a slightly higher percentage of DD cases in beef cattle but the greater percentages of sole lesions in dairy cattle and WLD being the same. Beef cattle also had a higher percentage of ‘other’ lesions. While these findings agree with the prevalence of lesions in beef cattle reported by UK foot trimmers [22], other work in UK beef cattle reported WLD, overgrowth and underrun sole as the most prevalent lesions [21]. It is very likely that some of the foot trimmers who contributed records to the present study also responded to the survey described by Fitzimmonds et al. [18], so similarities should be interpreted with caution. The available UK reports agree that WLD is an important lesion of beef cattle [21,22], while in Canada corkscrew claw and vertical fissures are the most observed lesion in beef cattle [35]. An analysis of the number of presentations of each foot showed that lesions in beef feet were, on average, presented fewer times than in dairy feet, with 63% of beef feet being observed to have lesions only once. This could be due to better lesion resolution in beef cattle but may also be due to the reported lower level of engagement between beef farms and foot trimmers [22].

Compared with similar studies from the UK, the number of lesions recorded in this data set was much higher than that recorded previously, including nearly 100,000 feet with compared with previous reports on 8600–14,200 lesions [13,15]. Previous analyses of large data sets of foot inspection records observed differences between operators using recording systems, even when using the same system [13,18]. The data set in this study was from foot trimmers working in the same organisation, which should lead to high consistency in recording practices. While all records coming from a single organisation improves consistency, it also biases the sample in terms of geography and predominant farming practices, meaning this sample of farms may not be representative of the wider UK. Regional variations in the prevalence of foot lesions were observed in previous studies [13]. A bias inherent to this data set is that only farms which undertake, and record, claw trimming are represented [18]. Previous studies have shown that lameness events predispose future lameness events in both heifers and cows [38,39,41], so any herd undertaking preventative trimming will have a different profile of foot lesions than herds not doing so [19]. Over half of UK dairy farms are reported as using external foot trimmers for preventative trimming [50] so, while imperfect, analysing foot inspection records should still be broadly representative.

## 5. Conclusions

This analysis of lesions of cattle feet confirmed that digital dermatitis, sole ulcer, sole haemorrhage and white line disease remain the most common lesions in cattle in the UK. The greatest change over 30 years was the increased prevalence of digital dermatitis, providing a key target for the UK cattle industry to address through farm protocols such as foot bathing. Most feet with lesions only appeared in the data set once, showing that most lesions were not re-presented and were likely resolved. More white line lesions were re-presented and had more severe grades compared with other lesions. The therapeutic trimming of chronic lesions appeared to be less successful, with re-presentations occurring, on average, every 49.5 days, compromising welfare for extended periods and requiring the consideration of different veterinary treatment options.

This study found that lesions were distributed among feet and claws, as described by the previous literature, but there was a significantly greater distribution of lesions on the right feet compared with the left feet, possibly linked to behavioural lateralisation. Although the number of beef cattle trimmed was very low, the foot lesions identified were similar to those of dairy cattle, with digital dermatitis being the most prevalent lesion identified.

## Figures and Tables

**Table 1:** Description of severity ratings of lesions used in all foot inspection records.

Severity	Description
Mild	Can be resolved with therapeutic trim or topical treatment only.
Moderate	Requires therapeutic trim/topical treatment and block/bandage.
Severe	Large or deep lesion requiring follow-up treatment.
Vet Advice	Lesion cannot be resolved adequately by trimmer. Requires veterinary attention.

**Table 2:** Summary of the number of feet with co-occurring major lesions recorded on them at first presentation (*n* = 1792). Number in brackets shows percentage of all foot records at first presentation.

Major lesion	Digital Dermatitis (*n*, %)	White Line Disease (*n*, %)	Sole Ulcer (*n*, %)	Sole Haemorrhage (*n*, %)
Digital Dermatitis	-	851 (1.6)	402 (0.8)	30 (0.06)
White Line Disease	851 (1.6)	-	33 (0.07)	44 (0.09)
Sole Ulcer	402 (0.8)	33 (0.07)	-	432 (0.86)
Sole Haemorrhage	30 (0.06)	44 (0.09)	432 (0.86)	-

*n* = number of feet.

**Table 3:** Numbers of lesions identified during therapeutic trimming, separated by foot.

	Left Hind	Right Hind	Left Fore	Right Fore	Left Feet (All)	Right Feet (All)
All records (major and minor lesions)	19,964	20,676	4774	4860	24,738	25,536
Digital dermatitis	6944	7364	898	867	7842	8231
Sole haemorrhage	2647	2810	519	471	3166	3281
Sole ulcer	3757	3984	963	958	4720	4942
White line disease	3953	3693	1437	1666	5390	5359

**Table 4:** Mixed effects model of the distribution of all lesions at first presentation (*n* = 50,276) between left and right feet. Left was represented as ‘0’ and right as ‘1’.

	Estimate	Standard Error	Z Value	*p*	Odds Ratio	95% Confidence Intervals
Intercept	0.031	0.011	2.78	0.005	1.03	1.01–1.06
**Random Effect**	**Variance**	**Standard Deviation**				
Farm	0.00023	0.003				
Trimmer	0.00034	0.018				
Lesion	0.00025	0.016				

Intercept shows the difference between left and right after controlling for other effects.

**Table 5:** Number of single major lesions recorded in different anatomical locations by foot and claw. Feet with multiple different lesions were excluded. A total of 36,253 lesion locations from 34,002 feet at their first presentation.

	Interdigital	Medial Claw	Lateral Claw
**All Feet**			
Digital Dermatitis	12,767	723	1035
Sole Haemorrhage	135	2607	3376
Sole Ulcer	152	3822	3561
White Line Disease	186	4028	3861
**Fore feet**			
Digital Dermatitis	1062	108	99
Sole Haemorrhage	7	731	488
Sole Ulcer	7	759	637
White Line Disease	22	1084	1027
**Hind feet**			
Digital Dermatitis	11,705	615	936
Sole Haemorrhage	77	1205	3529
Sole Ulcer	145	3063	2924
White Line Disease	154	2936	2827

**Table 6:** Mixed effects models of single major lesions identified at first presentation on either medial or lateral claws. Each disease has an overall model (denoted by ‘all feet’) and separate models for fore and hind feet. The intercept shows the difference remaining between medial and lateral claws once farm, trimmer and limb affected were accounted for. In all models, the medial claw is represented as 0 and the lateral claw as 1.

Intercept							Random Effects	
	Estimate	Standard Error	Z Value	*p*	Odds Ratio	95% Confidence Intervals	Variance	Standard Deviation
**Digital Dermatitis**								
All feet								
Intercept	0.52	0.23	2.24	0.03 *	1.69	1.05–2.77		
Farm							0.00077	0.0006
Trimmer							0.65224	0.8071
Limb							0.03544	0.1882
Fore feet								
Intercept	0.06	0.13	0.48	0.62	1.06	0.79–1.44		
Farm							0.04517	0.2125
Trimmer							0.00008	0.0006
Limb							0.00089	0.0002
Hind feet								
Intercept	0.65	0.28	2.35	0.01 *	1.92	1.02–3.78		
Farm							0.00007	0.0001
Trimmer							0.82571	0.9087
Limb							0.04822	0.2196
**Sole Haemorrhage**								
All feet								
Intercept	0.25	0.41	0.61	0.55	1.28	0.492–3.34		
Farm							1.0431	1.0213
Trimmer							0.7135	0.8447
Limb							0.4824	0.6945
Fore feet								
Intercept	−0.89	0.37	−2.41	0.02 *	0.412	0.184–0.864		
Farm							1.295	1.138
Trimmer							1.858	1.363
Limb							0.006	0.8
Hind feet	1.22	0.40	3.04	0.002 *	3.4	1.51–8.14		
Farm							2.1337	1.4761
Trimmer							2.6205	1.6188
Limb							0.0009	0.0296
**Sole Ulcer**								
All feet	0.16	0.18	0.92	0.36	1.18	0.816–1.69		
Farm							0.659	0.8118
Trimmer							0.3191	0.5649
Limb							0.0005	0.0009
Fore feet								
Intercept	−0.54	0.38	−1.41	0.16	0.585	0.26–1.27		
Farm							0.1814	1.3467
Trimmer							0.2159	1.4692
Limb							0.0004	0.0002
Hind feet								
Intercept	0.52	0.28	1.85	0.06	1.33	1.11–1.6		
Farm							1.5275	1.2359
Trimmer							1.2631	1.1239
Limb							0.0002	0.0143
**White Line Disease**								
All feet								
Intercept	0.15	0.11	1.39	0.16	1.16	0.936–1.46		
Farm							0.2597	0.5096
Trimmer							0.1503	0.3887
Limb							0.0009	0.0001
Fore feet								
Intercept	−0.22	0.22	−1.04	0.29	0.79	0.49–1.23		
Farm							0.3347	0.5786
Trimmer							0.6648	0.8154
Limb							0.0003	0.0005
Hind feet								
Intercept	0.48	0.28	1.71	0.08	1.62	0.92–2.95		
Farm							1.001	1
Trimmer							1.286	1.134
Limb							0.009	0.006

* indicates *p* < 0.05.

**Table 7:** Recorded severity of major lesions identified as single lesions at first presentation from 34,002 feet.

	Digital Dermatitis ^1^ (*n*, %)	Sole Haemorrhage ^2^ (*n*, %)	Sole Ulcer ^3^ (*n*, %)	White Line Disease ^4^ (*n*, %)
Mild	3158 (23.3)	553 (9.8)	1132 (15.8)	1022 (13.3)
Moderate	4705 (34.8)	2638 (46.7)	2997 (41.9)	2828 (36.8)
Severe	3989 (29.4)	1923 (34.1)	2281 (31.9)	2717 (35.3)
Vet Advice	1680 (12.4)	531 (9.4)	732 (10.2)	1116 (14.5)

*n* = number of feet. For total feet numbers for each lesion, ^1^ *n* = 13,532; ^2^ *n* = 5645; ^3^ *n* = 7142; ^4^ *n* = 7142.

**Table 8:** Trim frequencies and mean time between trims (to the nearest day) by foot of 50,276 feet from 32,557 cows on 346 farms.

Number of Trims	1	2	3	4	5	6	7	8	9	10+
Total	50,276	19,898	10,424	6112	3801	2437	1594	1106	765	1589
% of Total	51.3	20.3	10.6	6.2	3.9	2.5	1.6	1.1	0.8	1.6
Days from Previous Record	NA	142	219	123	145	165	126	147	159	174
Adjusted Days from Previous Record	NA	51	52	52	52	50	49	48	46	46

**Table 9:** Frequency and percentage of all recorded lesions at successive trim numbers of 50,276 feet from 32,557 cows on 346 farms.

Trim Number (*n*)	Digital Dermatitis (*n*, %)	Sole Ulcer (*n*, %)	Sole Haemorrhage (*n*, %)	White Line Disease (*n*, %)	Other Lesions (*n*, %)
1 (50,276)	16,073 (32.0)	9662 (19.2)	6447 (12.8)	10,785 (21.5)	7309 (14.5)
2 (19,898)	5545 (27.9)	4460 (22.4)	3098 (15.6)	4792 (24.1)	2003 (10.1)
3 (10,424)	2851 (27.4)	2509 (24.1)	1561 (15.0)	2604 (25.0)	899 (8.6)
4 (6112)	1391 (22.8)	1411(23.1)	883 (14.5)	1576 (25.8)	851 (13.9)
5 (3801)	783 (20.6)	906 (23.8)	501 (13.2)	1009 (26.6)	602 (15.8)
6 (2437)	470 (19.3)	545 (22.4)	360 (14.8)	619 (25.4)	443 (18.2)
7 (1594)	268 (16.8)	348 (21.8)	228 (14.3)	444 (27.9)	306 (19.2)
8 (1106)	197 (17.8)	239 (21.6)	156 (14.1)	296 (26.8)	218 (19.7)
9 (765)	135 (17.7)	159 (20.8)	107 (14.0)	207 (27.1)	157 (20.5)
10+ (1589)	225 (14.2)	330 (20.8)	216 (13.6)	394 (24.8)	424 (26.7)

*n* = number of feet. Percentages refer to percentage of all lesions at the given trim number.

**Table 10:** Percentages of 50,276 feet with lesions from 32,557 cows on 96 beef and 250 dairy farms.

Farm Type	Left Fore (%, *n*)	Right Fore (%, *n*)	Left Hind (%, *n*)	Right Hind (%, *n*)
Beef ^1^	9.0, (337)	8.3, (310)	40.2, (1510)	42.5, (1597)
Dairy ^2^	7.8, (7732)	8.19, (7718)	41.2, (38,761)	43.0, (40,477)

*n* = number of feet. For total numbers of feet by farm type, ^1^ *n* = 3754; ^2^ *n* = 94,190.

## Data Availability

The dataset presented in this article is not readily available for data protection reasons. Requests to access the datasets should be directed to the corresponding author.

## Appendix A

**Table A1:** Frequency, percentage and distribution of minor lesions at first presentation.

	No. Feet	% All Feet	Left Hind	Right Hind	Left Fore	Right Fore
Abscess—Not White Line	189	0.38%	76	68	12	33
Acute Laminitis	8	0.02%	4	3	1	0
Axial Wall Fissure	252	0.50%	87	93	29	43
Corkscrew Claw	320	0.64%	69	84	86	81
Double Sole	5550	11.04%	1983	1996	720	851
Horizontal Cracks	84	0.17%	32	22	16	14
Vertical Cracks	415	0.83%	135	126	66	88
Foreign Body	1322	2.63%	339	374	286	323
Interdigital Phlegmon	1404	2.79%	565	582	127	257
Heel Erosion	15	0.03%	5	9	1	0
Heel Ulcer	1213	2.41%	509	515	88	101
Hoof Avulsion/Injury	46	0.09%	16	21	5	4
Hoof Wall Lesion	99	0.20%	44	36	12	7
Infected Joint/Tendon Sheath	83	0.17%	31	35	9	8
Interdigital Hyperplasia	1856	3.69%	847	893	69	47
Laminitis	28	0.06%	11	11	3	3
Overgrown	113	0.22%	40	45	13	15
Pus	1464	2.91%	496	479	229	260
Shallow Heel	7	0.01%	3	4	0	0
Thin Sole	994	1.98%	348	370	133	143
Toe Necrosis	1078	2.14%	403	439	130	106
Underrun Heels	33	0.07%	14	12	4	3

All minor lesions were more likely to be reported in hind feet, with the exception of corkscrew claw.

## Appendix B

**Table A2:** Percentages and odds ratio of repeated lesions.

Trim Number	Digital Dermatitis		Sole Ulcer		Sole Haemorrhage		White Line Disease	
	%	Odds Ratio (95%CI)	%	Odds Ratio (95%CI)	%	Odds Ratio (95%CI)	%	Odds Ratio (95%CI)
2	68.01%	2.89 (2.72, 3.08)	55.40%	3.69 (3.45, 3.94)	28.08%	1.96 (1.80, 2.14)	62.46%	4.19 (3.93, 4.48)
3	58.12%	3.90 (3.58, 4.25)	55.16%	4.99 (4.55, 5.48)	29.66%	2.43 (2.15, 2.75)	66.24%	7.01 (6.38, 7.70)
4	61.97%	5.27 (4.66, 5.96)	59.74%	6.35 (5.61, 7.20)	33.52%	3.89 (3.31, 4.57)	67.51%	9.18 (8.09, 10.39)
5	59.77%	7.37 (6.24, 8.72)	54.19%	6.78 (5.77, 7.97)	34.93%	4.64 (3.75, 5.73)	70.07%	11.53 (9.80, 13.56)
6	57.66%	8.56 (6.88, 10.65)	55.05%	6.41 (5.24, 7.86)	36.11%	8.33 (6.33, 10.96)	71.24%	11.55 (9.40, 14.18)
7	58.96%	9.43 (7.09, 12.54)	48.28%	5.90 (4.56, 7.65)	42.11%	7.53 (5.48, 10.34)	69.59%	15.04 (11.63, 19.45)
8	50.25%	12.93 (8.94, 18.71)	50.21%	8.02 (5.81, 11.07)	38.46%	8.25 (5.52, 12.32)	73.31%	15.03 (11.01, 20.53)
9	54.81%	12.32 (8.04, 18.90)	55.97%	9.63 (6.52, 14.23)	38.32%	8.34 (5.13, 13.56)	71.01%	15.36 (10.56, 22.33)
10	60.47%	13.64 (8.08, 23.03)	50.50%	7.42 (4.61, 11.96)	45.59%	9.78 (5.48, 17.43)	72.19%	17.24 (10.96, 27.14)

Odds ratios show the odds of a lesion being recorded if the previous trim had the same lesion.
